# RCT versus real-world cohorts: Differences in patient characteristics drive associations with outcome after EVT

**DOI:** 10.1177/23969873221142642

**Published:** 2022-12-16

**Authors:** Fanny Quandt, Nina Meißner, Teresa A Wölfer, Fabian Flottmann, Milani Deb-Chatterji, Lars Kellert, Jens Fiehler, Mayank Goyal, Jeffrey L Saver, Christian Gerloff, Götz Thomalla, Steffen Tiedt

**Affiliations:** 1 Department of Neurology, University Medical Center Hamburg-Eppendorf, Hamburg, Germany; 2 Institute for Stroke and Dementia Research, University Hospital, LMU Munich, Munich, Germany; 3 Department of Diagnostic and Interventional Neuroradiology, University Medical Center Hamburg-Eppendorf, Hamburg, Germany; 4 Department of Neurology, University Hospital, LMU Munich, Munich, Germany; 5 Department of Radiology, University of Calgary, Foothills Medical Centre, Calgary, AB, Canada; 6 Department of Neurology and Comprehensive Stroke Center, David Geffen School of Medicine, University of California Los Angeles, Los Angeles, CA, USA

**Keywords:** Stroke, Endovascular thrombectomy, real-world data, RCT, outcome prediction

## Abstract

**Background:**

The selection of patients with large-vessel occlusion (LVO) stroke for endovascular treatment (EVT) depends on patient characteristics and procedural metrics. The relation of these variables to functional outcome after EVT has been assessed in numerous datasets from both randomized controlled trials (RCT) and real-world registries, but whether differences in their case mix modulate outcome prediction is unknown.

**Methods:**

We leveraged data from individual patients with anterior LVO stroke treated with EVT from completed RCTs from the Virtual International Stroke Trials Archive (*N* = 479) and from the German Stroke Registry (*N* = 4079). Cohorts were compared regarding (i) patient characteristics and procedural pre-EVT metrics, (ii) these variables’ relation to functional outcome, and (iii) the performance of derived outcome prediction models. Relation to outcome (functional dependence defined by a modified Rankin Scale score of 3–6 at 90 days) was analyzed by logistic regression models and a machine learning algorithm.

**Results:**

Ten out of 11 analyzed baseline variables differed between the RCT and real-world cohort: RCT patients were younger, had higher admission NIHSS scores, and received thrombolysis more often (all *p* < 0.0001). Largest differences at the level of individual outcome predictors were observed for age (RCT: adjusted odds ratio (aOR), 1.29 (95% CI, 1.10–1.53) vs real-world aOR, 1.65 (95% CI, 1.54–1.78) per 10-year increments, *p* < 0.001). Treatment with intravenous thrombolysis was not significantly associated with functional outcome in the RCT cohort (aOR, 1.64 (95 % CI, 0.91–3.00)), but in the real-world cohort (aOR, 0.81 (95% CI, 0.69–0.96); *p* for cohort heterogeneity = 0.056). Outcome prediction was more accurate when constructing and testing the model using real-world data compared to construction with RCT data and testing on real-world data (area under the curve, 0.82 (95% CI, 0.79–0.85) vs 0.79 (95% CI, 0.77–0.80), *p* = 0.004).

**Conclusions:**

RCT and real-world cohorts considerably differ in patient characteristics, individual outcome predictor strength, and overall outcome prediction model performance.

## Introduction

Whether and how patients with large-vessel occlusion (LVO) stroke benefit from endovascular treatment (EVT) depends on patient characteristics such as age^1,2^ and comorbidities^3^ as well as procedural metrics including time intervals.^4^ The relation of these variables to functional outcome after EVT drives the selection of patients for EVT, the design of clinical pathways as well as of randomized controlled trials (RCTs). To investigate the value of an individual predictor or to construct an outcome prediction model, numerous datasets have been leveraged derived both from RCTs^4–6^ as well as prospective real-world registries.^7–9^ While RCTs usually apply stringent inclusion and exclusion criteria for patient recruitment to increase the likelihood to detect a treatment effect, prospective registries depict real-world data including patient groups underrepresented in RCTs. Differences in the case mix between RCT and real-world cohorts are widely acknowledged,^10^ however, whether these also influence the relation of individual predictors with outcome or the performance of derived outcome prediction models has not been assessed in depth. Knowledge of differences and similarities between datasets would facilitate the interpretation of the plethora of studies on outcome prediction.

Here, we systematically compared individual patient-level data from RCTs on EVT and a large real-world cohort of patients with stroke undergoing EVT with regards to (i) patient characteristics at baseline and procedural pre-EVT metrics, (ii) the relation of these variables to functional outcome, and (iii) the performance of derived outcome prediction models.

## Methods

Findings of this study were derived from data obtained from the Virtual International Stroke Trials Archive (VISTA) and the German Stroke Registry (GSR). The availability of these data is restricted and was specifically issued for the current study. These data are not publicly available. The R-script for the statistical analyses can be obtained from the first author upon reasonable request.

### Study samples

#### Virtual International Stroke Trials Archive – Endovascular

**Figure 1.**
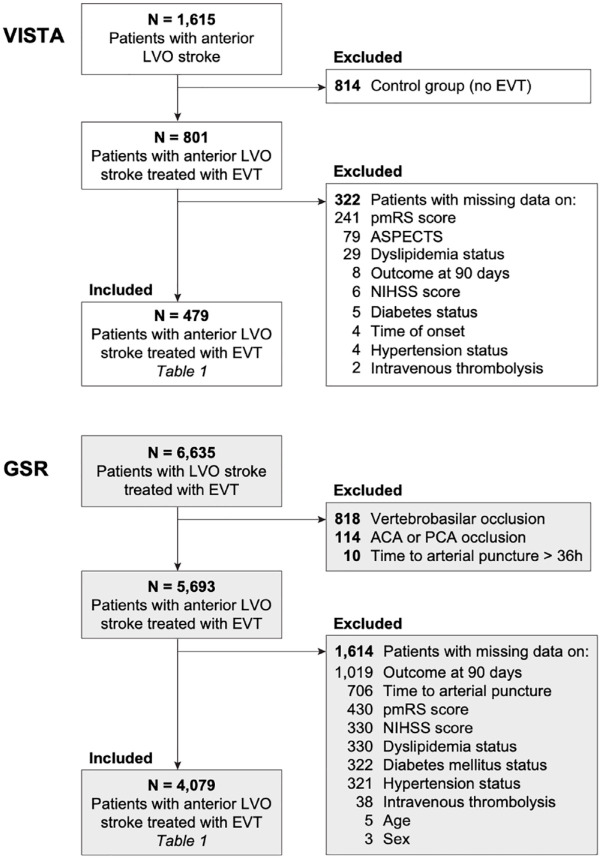
Flowchart of patients with LVO stroke included into analyses. GSR: German Stroke Registry; VISTA: Virtual International Stroke Trials Archive; LVO: large-vessel occlusion; EVT: endovascular treatment; pmRS: premorbid modified Rankin Scale; NIHSS: National Institutes of Health Stroke Scale; PCA: posterior cerebral artery; ACA: anterior cerebral artery; ΔT: time difference; ASPECTS: Alberta Stroke Program Early CT Score; h: hour.

The Endovascular subsection of VISTA (VISTA-Endovascular)^11^ encompasses data from RCTs meeting the following criteria: *N* ⩾ 20 participants, baseline assessment including information on neurological impairment and any type of vessel imaging conducted within 24 h of stroke onset, and functional outcome assessed using the modified Rankin Scale (mRS) score at 90 days after stroke onset. From VISTA-Endovascular we retrieved data from completed RCTs comprising overall 1615 patients with ischemic stroke due to LVO in the anterior circulation treated with EVT or best medical care. We excluded 1136 patients that were part of the control group without EVT or lacked information on any of the included variables (Figure 1, Table 1).

**Table 1.** Characteristics of patients with anterior circulation LVO stroke that underwent EVT from the German Stroke Registry and from VISTA-Endovascular.

Characteristics	GSR	VISTA	*p* Value
	*N* = 4079	*N* = 479	
Age, median (IQR) (years)	76 (65–82)	68 (57–78)	<0.0001
Female, *N* (%)	2098 (51.4)	223 (46.6)	0.048
Medical history, *N* (%)			
Hypertension	3161 (77.5)	265 (53.2)	<0.0001
Diabetes mellitus	898 (22.0)	79 (16.5)	0.005
Dyslipidemia	1655 (40.6)	136 (28.4)	<0.0001
Current smoking	567 (16.9)	136 (28.4)	<0.0001
Pre-stroke mRS score, median (IQR)	0 (0–1)	0 (0–0)	<0.0001
Baseline NIHSS score, median (IQR)	15 (10–18)	17 (13–21)	<0.0001
ASPECTS, *N* (%)			0.047
High (9–10)	1980 (54.2)	262 (54.7)	
Middle (6–8)	1343 (36.8)	189 (39.5)	
Low (0–5)	330 (9.0)	28 (5.8)	
Intravenous thrombolysis, *N* (%)	2187 (53.6)	407 (85.0)	<0.0001
Symptom onset to arterial puncture, median (IQR) (min)	220 (153–334)	232 (172–290.5)	0.743
mRS score 0–2 at 90 days after stroke, *N* (%)	1570 (38.7)	215 (44.9)	0.009

IQR: interquartile range.

Symptom onset to arterial puncture was imputed in 41% of VISTA-Endovascular patients (see methods section).

#### German Stroke Registry – Endovascular Treatment

The German Stroke Registry – Endovascular Treatment (GSR, ClinicalTrials.gov Identifier: NCT03356392) is an ongoing, academic, prospective, multicenter registry in Germany^9^ and includes patients with a diagnosis of acute ischemic stroke due to LVO, initiation of EVT, and age ⩾18 years without any exclusion criteria. We retrieved data from 6635 patients, which were recruited between 2015 and 2019 at 25 centers distributed across Germany. We excluded 2556 patients that had occlusions of the vertebrobasilar circulation or exclusively of the anterior or posterior cerebral artery, had an onset-to-puncture time >36 h or that lacked information on any of the included variables (Figure 1, Table 1). The study was approved both centrally by the Institutional Review Board of the Ludwig-Maximilians-Universität Munich (protocol No 689-15) as well as by local review boards and was conducted in accordance with the Declaration of Helsinki.

#### RCT cohort subsets

Inclusion and exclusion criteria of five RCTs were listed and screened for whether (i) the underlying information is available in the GSR and (ii) they differ from selection criteria of the GSR (Supplemental Table 3). From these selected criteria (Table 2), we chose three common criteria (NIHSS score >5, time from onset to puncture <12 h, pmRS score <2) to be applied to the GSR and VISTA dataset to construct RCT-like subsets. These subsets were compared using similar approaches as for the unrestricted datasets as described below.

**Table 2.** Patients from the German Stroke Registry matching RCT selection criteria.

RCT	Threshold-based exclusion of GSR patients						Included GSR patients
	pmRS	Age	Time*	NIHSS	Vessel	ASPECTS	
MR CLEAN, *N* (%)	–	–	⩽6 h, 905 (22.2)	⩾2, 60 (1.5)	dICA, M1/2, A1/2, 498 (12.2)	–	2876 (70.5)
ESCAPE, *N* (%)	⩽2**, 482 (11.8)	–	⩽12 h***, 138 (3.4)	>5, 392 (9.6)	dICA, M1, 1052 (25.8)	>5, 330 (8.1)	2185 (53.6)
SWIFT-PRIME, *N* (%)	⩽1, 866 (21.2)	18–80, 1328 (32.6)	⩽6 h****, 905 (22.2)	9–29, 888 (21.8)	ICA, M1, 792 (19.4)	–	1230 (30.2)
EXTEND-IA, *N* (%)	⩽1, 866 (21.2)	–	⩽6 h, 905 (22.2)	–	–	–	2515 (61.7)
REVASCAT, *N* (%)	⩽1, 866 (21.2)	18–80, 1328 (32.6)	⩽8 h, 905 (22.2)	>5, 392 (9.6)	dICA, M1, 1052 (25.8)	>5, 330 (8.1)	1301 (31.9)

RCT: randomized controlled trial; GSR: German Stroke Registry; dICA: distal ICA.

Threshold-based exclusion of GSR patients was performed for RCT selection criteria, for which information was available in the GSR and which differed from GSR selection criteria.

* Time from onset to arterial puncture.

** The study criterion was adapted by substituting the Barthel-threshold (90) with a pmRS score.

*** The study criterion was adapted by substituting the time of randomization with the time of arterial puncture.

**** The study criterion was adapted by substituting the onset-to-rtPA- and CTP-to-treatment threshold (4.5 h + 1.5 h) with an onset to arterial puncture threshold (6 h).

### Outcome measures

In both datasets functional outcome was assessed at 90 days using the mRS score ranging from 0 (no symptoms) to 6 (death). The primary outcome was functional independence (mRS 0–2) at 90 days. Secondary outcome measures included excellent functional outcome (mRS 0–1) and moderate functional outcome (mRS 0–3).

### Predictor variable selection and data preprocessing

From both datasets, VISTA-Endovascular and GSR, we selected all variables that were available (i) prior to arterial puncture and (ii) in both datasets. We excluded variables with more than 5% missing values except for, owing to their clinical importance, the pre-stroke modified Rankin Scale (pmRS) score (31% missing values in VISTA-Endovascular) and the time from symptom onset to arterial puncture and ASPECTS (10% missing values each in the GSR), overall resulting in 11 variables (Table 1). We grouped ASPECTS into not available, low (0–5), middle (6–8), and high (9–10). Time from symptom onset to arterial puncture was not available in 41% of patients from VISTA-Endovascular: for these patients, we imputed the time from symptom onset to arterial puncture by adding 0.71 h, the average difference between the time of randomization and the time of arterial puncture in patients with both available times, to the time from onset to randomization.

### Statistical and machine learning analyses

All analyses were performed in “R,” version 4.1.2.

#### Conventional statistical analysis

Differences in patient characteristics and procedural metrics of the study samples were assessed using the Mann–Whitney test, the Fisher’s exact test, and linear and logistic regression as appropriate. Differences in probability distributions were assessed using the Kolmogorov-Smirnov-Test. To determine the relation between baseline parameters and functional outcome at 90 days after stroke, we performed unadjusted and adjusted logistic regression. Continuous variables were not scaled. Adjusted analyses included all 11 variables in the model. Odds ratios are presented in their relation to functional dependence. Differences of the relation of specified variables with the primary outcome (mRS score 0–2 at 90 days) between cohorts were tested using a multiplicative interaction term (cohort × specified variable) in adjusted logistic regression models.

#### Machine learning and predictive models

Machine learning models for outcome prediction included gradient boosting machines (GBM), extreme gradient boosting, support vector machines, random forests, and logistic regression models using the “R” package “caret” version 6.0.86.^12^ Throughout the analyses, data were randomly split into a training set (80%) and a test set (20%). Observing uneven distribution of outcomes, we down-sampled data within each fold to avoid classification bias. The model was trained with a repeated (*n* = 100) 5-fold cross-validation within the training set. We optimized hyperparameters using a grid search algorithm to improve the area under the curve (AUC) within the training set (independently for VISTA and GSR models). We determined discriminative model performances in the test set and quantified them as the AUC of the receiver operating characteristic (ROC). We used bootstrap replicates to calculate 95% confidence intervals of ROC curves and the Delong method (“R” package “pROC” version 1.16.2)^13^ to compare ROC curves. Model calibration, assessing the level of agreement between predicted and observed probability, was assessed graphically with a plot for the prediction of functional dependence and quantified by the intercept and slope. Further, we calculated model-based concordance^14^ to quantify the expected change in model performance due to case-mix heterogeneity.

#### Variable importance for outcome prediction

The value of a variable for the prediction of functional outcome was the change in model performance after permuting this variable a hundred times so that a larger drop in performance would correspond to higher variable importance. We applied variable importance analyses from the “R” package “iml” version 0.10.0^15^ and used the full datasets for these analyses.

## Results

**Figure 2.**
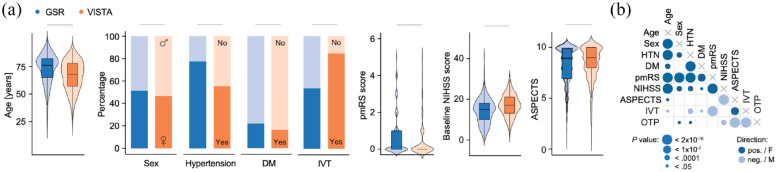
Differences in baseline characteristics between real-world patients and patients included in RCTs. (a) Real-world patients (GSR) were older and more likely to present with hypertension and diabetes and had higher pmRS and lower NIHSS scores and ASPECTS upon admission compared to the RCT cohort (VISTA). The distribution of age, pmRS, and NIHSS scores was significantly different between the GSR and VISTA cohorts. (b) The majority of baseline characteristics were significantly associated with each other (26 of 36 investigated associations). (a) Wilcoxon test and Fisher’s exact test. Horizontal bars indicate *p* < 0.05 for group differences. Dashed horizontal bars indicate *p* < 0.05 for different probability distributions (Kolmogorov-Smirnov-Test). (b) Mann–Whitney test, Fisher’s exact test, and logistic or linear regression models as appropriate. Circle size indicates *p* value categories, circle color indicates the direction of the relationship. Data from the GSR. GSR: German Stroke Registry; VISTA: Virtual International Stroke Trials Archive; DM: diabetes mellitus; IVT: intravenous thrombolysis; pmRS: premorbid modified Rankin Scale; NIHSS: National Institutes of Health Stroke Scale; ASPECTS: Alberta Stroke Program Early CT Score; HTN: hypertension; OTP: onset to puncture time; pos.: positive; neg.: negative; F: female; M: male.

We analyzed data from individual patients with anterior circulation LVO stroke treated with EVT and either recruited to RCTs on EVT efficacy (*N* = 479; from the Endovascular subsection of the Virtual International Stroke Trials Archive (VISTA-Endovascular)) or included into a real-world registry (*N* = 4079; from the German Stroke Registry (GSR); Figure 1). The percentage of patients with good functional outcome was higher in the RCT cohort (VISTA-Endovascular) compared to the real-world cohort (GSR, 44.9% vs 38.7%, *p* = 0.009, Table 1). Ten out of 11 baseline variables were significantly different between the RCT and real-world cohort: compared to patients treated in real-world, RCT patients were less likely to be female (47% vs 51%, *p* = 0.048), were younger (68 vs 76 years, *p* < 0.0001), were less likely to present with hypertension (53% vs 78%, *p* < 0.0001), diabetes (17% vs 22%, *p* = 0.005), and dyslipidemia (28% vs 41%, *p* < 0.0001), were more likely to currently smoke (28% vs 17%, *p* < 0.0001), had lower pmRS scores (*p* < 0.0001), but higher NIHSS scores (17 vs 15, *p* < 0.0001) and higher ASPECTS (*p* = 0.047) upon admission, and received intravenous thrombolysis more often (85% vs 54%, *p* < 0.0001, Table 1, Figure 2(a)). Both cohorts also differed in their probability distributions for age, pmRS and NIHSS scores (Figure 2(a), all *p* < 0.0001). Most baseline variables were significantly associated with each other (26 of 36 analyzed associations, Figure 2(b)). The percentage of patients treated in real-world that fulfilled the specified selection criteria (both inclusion and exclusion criteria) from five RCTs on EVT that were published in 2015 prior to recruitment of the first real-world patient ranged from 30.2% to 70.5% (Table 2, Supplemental Table 3).

### Relation of baseline variables to functional outcome

**Figure 3.**
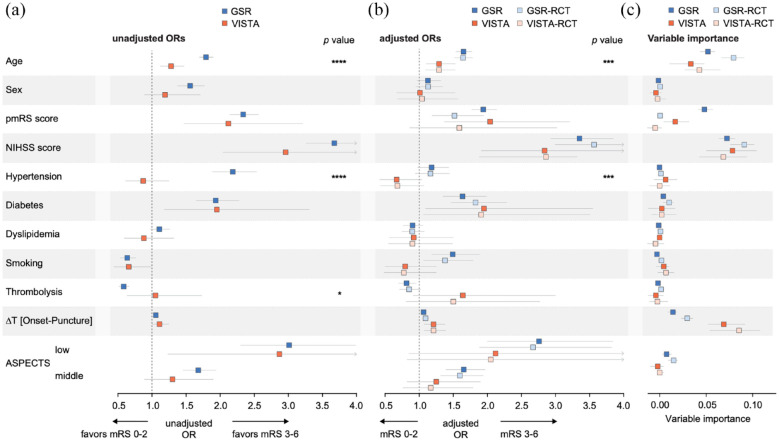
Association of patient characteristics and other metrics with functional outcome in real-world patients and patients included in RCTs. (a) Age, hypertension, and treatment with intravenous thrombolysis significantly differed in their relation to functional outcome between both cohorts in unadjusted analyses. (b) Age and hypertension significantly differed in their relation to functional outcome between both cohorts also in adjusted analyses. GSR and VISTA subsets that aligned with common RCT selection criteria (NIHSS >5, time from symptom onset to groin puncture <12 h, pmRS < 2) showed similar differences as the unrestricted cohorts. (c) Age, the pmRS score, and onset-to-puncture time showed different variable importance for predicting outcome in real-world (GSR) and RCT data (VISTA). (a and b) Univariable (a) and multivariable (b) logistic regression analyses. Multivariable analyses were adjusted for all other variables. Odds ratios were calculated for age per 10-year increments, for female versus male sex, for the pmRS score in one-point increments, for the NIHSS score in 10-point increments, for hypertension, diabetes, and dyslipidemia with yes versus no, for smoking with any history of smoking versus no history, for thrombolysis with treatment versus no treatment, for the time from onset to puncture per 1-h delay, and for ASPECTS with low (0–5) and middle (6–8) versus high. *p* Values indicate the levels of significance from the interaction term for investigating whether cohort assignment (RCT vs RWD) introduces heterogeneity and are indicated as follows: * for *p* < 0.05, *** for *p* < 0.001, **** for *p* < 0.0001. (c) Variable importance analyses to calculate the contribution of each parameter to outcome prediction using a machine learning algorithm (gradient boosting machines). Boxes indicate the median and whiskers the 5% and 95% quantiles. (a–c) Data from the GSR cohort (*N* = 4079), the VISTA cohort (*N* = 479), the GSR-RCT cohort (*N* = 2772), and the VISTA-RCT cohort (*N* = 446).

We next investigated whether the observed differences across baseline variables would extend to how these variables are related to outcome: in unadjusted analyses, we found significant differences between both cohorts for age, hypertension (both *p* < 0.0001), and treatment with intravenous thrombolysis (*p* = 0.03, Figure 3(a)). Analyses adjusted for all other variables showed significant differences between cohorts for age and hypertension (both *p* < 0.001): while hypertension was not significantly associated with functional dependence in any of the cohorts (*p* > 0.05), a 10-year increment of age was associated with 1.29 times higher odds (95% confidence interval (95% CI) of adjusted odds ratio (aOR), 1.10–1.53) for functional dependence in the RCT cohort and 1.65 times higher odds (95% CI of aOR, 1.54–1.78) in the real-world cohort. Of note, treatment with intravenous thrombolysis was not significantly associated with functional outcome in the RCT cohort (aOR, 1.64 (95% CI, 0.91–3.00)), but in the real-world cohort (aOR, 0.81 (95% CI, 0.69–0.96), *p* for heterogeneity between cohorts = 0.056). A 1-h increment of the time from onset to arterial puncture was associated with a 1.21 times higher odds of functional dependence at 90 days (95% CI of aOR, 1.07–1.38) in the RCT cohort and 1.06 times higher odds (95% CI of aOR, 1.04–1.09) in the real-world cohort (*p* = 0.27). Similar results were obtained when restricting both datasets to patients with a time from onset to arterial puncture of <6 h (Supplemental Figure 3). To investigate whether the observed differences would be caused by more specific patient selection criteria in RCTs, we restricted the real-world cohort and the RCT cohort to patients complying with common RCT inclusion criteria (NIHSS score >5, time from symptom onset to groin puncture <12 h, pmRS score <2; Table 2, Supplemental Table 3)^1^ resulting in cohorts of 2772 GSR patients (68.0% of total GSR cohort) and 446 VISTA patients (93.1% of the total VISTA cohort): of note, we found that differences between most variables were preserved, both between baseline characteristics (Supplemental Table 4) and for associations with outcome (Figure 3(b)).

To determine whether the different relation of baseline variables to outcome between both cohorts would extend to the contribution of these variables to outcome prediction model performance, we applied variable importance analyses: the NIHSS score upon admission was the most important variable for outcome prediction in both datasets. Considerable differences between datasets were seen for the time from onset to arterial puncture time, age, and the pmRS score: while time from onset to arterial puncture was the second most important variable in the RCT dataset but much less important in the real-world dataset, age, and the pmRS score were less important in RCT compared to real-world data (Figure 3(c)), overall in line with the observed differences between odds ratios. Of note, variable importance for outcome prediction was not related to variable variance (Supplemental Figure 1).

### Performance of outcome prediction models

**Figure 4.**
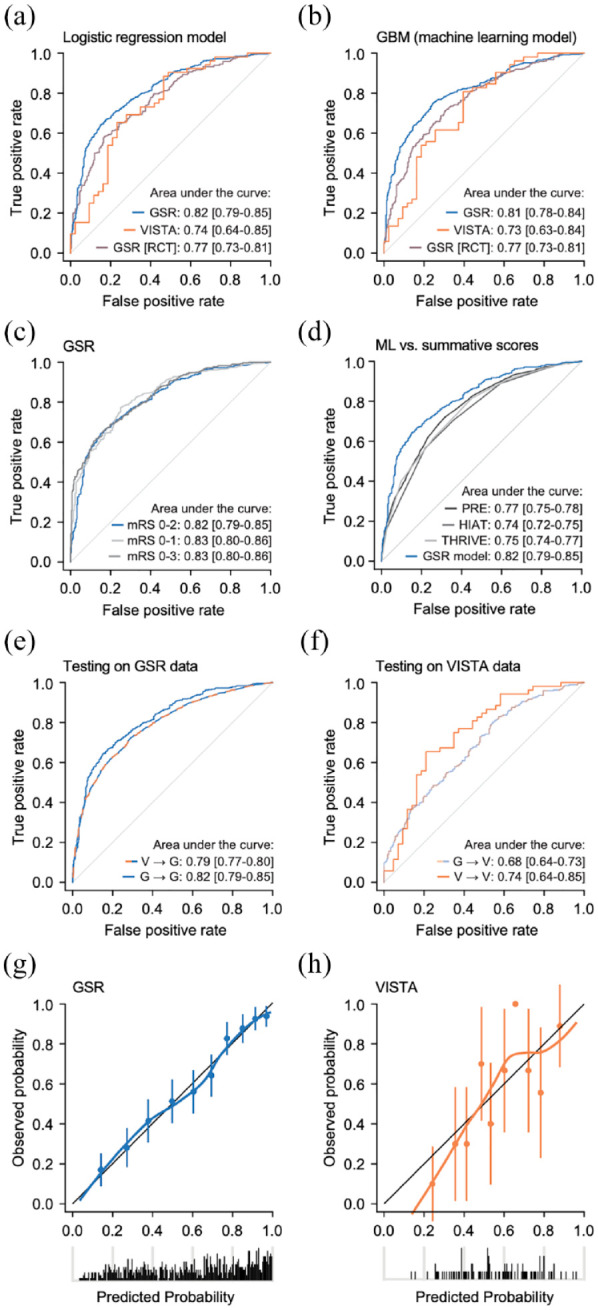
Outcome prediction using real-world and RCT data. (a and b) Real-world data (GSR) were more precise than RCT data (VISTA) in predicting outcome both when using a logistic regression model (a) and a machine learning algorithm (b). Restricting the real-world cohort to RCT-like patients revealed an intermediate AUC. (c) Outcome prediction was independent of how functional outcome was dichotomized. (d) This model outperformed previously reported summative scores. (e) Testing the RCT model on real-world data revealed a lower AUC compared to when testing the real-world-derived model on real-world data. (f) Testing the real-world-derived model on an RCT cohort revealed a smaller AUC compared to when testing the RCT-derived model on RCT data. (g and h) Real-world data model showed superior calibration compared to RCT data model. (a, c–h) Multivariable logistic regression model. (b) Machine learning algorithm (gradient boosting machines). (a–h) Data from the GSR cohort, the VISTA cohort, and the GSR-RCT cohort (NIHSS >5, time from symptom onset to puncture <12 h, pmRS score <2). G: GSR; V: VISTA; ML: machine learning; OTP: time from onset to puncture.

Lastly, we compared the performance of outcome prediction models derived from both cohorts. In a logistic regression model, pre-arterial puncture variables predicted functional outcome in the RCT cohort with an AUC of 0.74 (95% CI, 0.64–0.85), which was numerically but not significantly lower compared to the AUC achieved using real-world data (AUC, 0.82 (95% CI, 0.79–0.85), *p* = 0.18). The model from real-world data showed superior calibration compared to RCT data (intercept = 0.035, slope = 0.929 vs intercept = 0.079, slope = 1.136) (Figure 4(g) and (h)). Restricting the real-world cohort to patients complying with RCT inclusion criteria revealed an intermediate performance (AUC, 0.77 (95% CI, 0.73–0.81), Figure 4(a)). Considering the high degree of correlation between variables (Figure 2(b)), we also evaluated different machine learning algorithms, which are characterized by a non-linear structure and thus offer an alternative approach in settings of high-level interdependencies and multidimensional patterns.^16^ Gradient boosting machines showed the highest value in predicting functional outcome of patients with anterior circulation LVO stroke (Supplemental Figure 2(a)) and were subsequently further advanced by optimizing the hyperparameters (Supplemental Figure 2(b)). Using this algorithm, we found similar results as with logistic regression: accuracies in the RCT dataset (AUC, 0.73 (95% CI, 0.63–0.84)) and the RCT-like real-world dataset (AUC, 0.77 (95% CI, 0.73–0.81)) were lower than in the real-world dataset (AUC, 0.81 (95% CI, 0.78–0.84), Figure 4(b)). We thus continued with the logistic regression model and found model performance in the real-world dataset independent of how functional outcome was dichotomized (Figure 4(c)) and superior to the previously reported summative scores PRE,^17^ HIAT2,^18^ and THRIVE^19^ (all *p* < 0.005, Figure 4(d)). Lastly, we assessed how an RCT-derived model would inform on real-world outcomes: testing the RCT classifier on real-world data revealed an AUC of 0.79 (95% CI, 0.77–0.82), which was lower compared to the real-world-derived classifier (*p* = 0.004, Figure 4(e)). Model-based concordance confirmed that the difference of model performance in the RCT-derived model is driven by case-mix heterogeneity in real-world data, indicated by an increase in the *mbc* in the external validation data (real-world data, *mbc* = 0.80) compared to the *mbc* in the developmental data (RCT data, *mbc* = 0.73). In turn, testing the real-world classifier on RCT patients revealed an AUC of 0.68 (95% CI, 0.64–0.73, *mbc* = 0.77), which was lower than the RCT-derived classifier (AUC, 0.74 (95% CI, 0.64–0.85), *mbc* = 0.83, Figure 4(f)).

## Discussion

Cumulating data from almost 5000 patients with anterior circulation LVO stroke treated with EVT, we found considerable differences between an RCT-derived cohort and a large real-world cohort with regards to patient characteristics and procedural metrics, the relation of these variables with functional outcome, and the performance of outcome prediction models: (i) RCT patients were younger and less premorbid, but were more affected by stroke and more often treated with intravenous thrombolysis; (ii) the odds of functional dependence at 90 days were significantly lower for RCT patients compared to real-world patients per 10-year increment of age, while treatment with intravenous thrombolysis was not associated with outcome in RCT patients but in real-world patients; and (iii) outcome prediction tested on real-world patients was more informative when training the model in a real-world cohort compared to an RCT cohort.

Whether and how individual variables relate to outcome drives clinical decisions and RCT design. Such data are leveraged from different real-world registries as well as RCTs and are often reported side by side. Previous studies comparing real-world registry- and RCT-derived data were either constrained by analyzing cumulative and not individual patient-level data or were focused on the comparison of functional outcomes and adverse effects^20,21^ and did not include a comparison of individual predictors. Our analyses point toward considerable differences between RCT and real-world cohorts in how variables such as age and time from onset to treatment initiation are related to outcome. We further found that a real-world cohort complying with RCT selection criteria showed intermediate odds ratios for most variables indicating that other factors than lower and upper limits for variables might be important, for example, the distribution of patients within RCT limits. Further, the observed differences might be explained by variables being differently important for outcome between subgroups with different prevalence between cohorts. From a precision medicine perspective that logic might be extended to the individual patient, meaning that there is not one true biological association of a specific trait with outcome but that the influence of each variable on outcome is different for every patient. Considering this and the growing number of research studies analyzing an increasing number of different cohorts, our data call for the consideration of baseline patient characteristics and procedural metrics when interpreting the association of individual variables with outcome of one specific study. Interpreting differences and similarities between studies leveraging data from different cohorts would be facilitated by reducing methodological differences as much as possible, for example, by instructing researchers to integrate results from a model adjusted for one list of variables available in every dataset. Another option to increase awareness and transparency might be the obligation to compare baseline patient characteristics to a standard dataset openly available.

Our data are in line with and extend results from previous studies on the association of patient characteristics and procedural metrics with functional outcome. Importantly, we found a similar relation between time from onset to EVT start with outcome as the HERMES study group, which observed 0.81 times lower odds of functional independence per 1-h delay to reperfusion in 634 RCT patients.^4^ In contrast to our findings on a weaker association between time from onset to EVT start and outcome in real-world patients compared to RCT patients, the MR CLEAN Registry Investigators observed a stronger association between time from onset to EVT start and outcome in real-world patients (*N* = 1488, aOR, 0.78 (95% CI, 0.70–0.86))^22^ when compared to RCT patients. These differential findings might be routed in differences of the case mix between the MR CLEAN Registry (e.g. median age 71 years) and the GSR (median age 76 years) as well as of inclusion criteria: the MR CLEAN Registry analysis included only patients with time to arterial puncture within 6.5 h of stroke onset, while no such time criterion was used during recruitment to the GSR. Considering that the magnitude of the association between time to EVT and outcome has been reported to be larger in patients well-suited for EVT,^4^ the MR CLEAN Registry study might thus have selected patients with higher benefit from EVT compared to the GSR. Importantly, we found similar differences of the importance of time to treatment for outcome prediction between the real-world and VISTA datasets when restricting the cohorts to patients with a time from onset to arterial puncture of less than 6 h.

Further, it is important to note that our findings evidenced a differential association of treatment with intravenous thrombolysis with outcome between RCT and real-world patients, which is of interest in light of the ongoing discussion on the added value of intravenous thrombolysis in EVT treated patients. Our findings, however, need to be interpreted with care considering that the number of RCT patients not receiving intravenous thrombolysis in our study was low.

Outcome prediction models might support the selection of patients for treatment, including of stroke patients for EVT.^23^ Here, we found highest performance when training and testing a machine learning model on real-world data. While RCTs collect high-quality data, they are in most cases highly selective when recruiting patients. In contrast, real-world registries include patient subgroups excluded from or underrepresented in RCTs, but compile data that is usually of lower quality and less complete. Our findings indicate that the inclusion of such subgroups into the training set is important to predict outcome across the whole range of real-world patients. Our data thus support the notion that, if developed with the goal of real-world implementation, such models should also be developed using real-world data.

Strengths of our study include the combination of individual patient-level data from both multiple RCTs as well as a real-world registry leveraging an overall large sample size and the application of both conventional statistics as well as machine learning algorithms. Limitations of our study include potentially underlying biases including of patient selection in real-world data, which might have influenced our results, but which are inherent to real-world data. Also, we cannot exclude that differences in clinical pathways for RCT patients in comparison to real-world registry patients might account for some of the observed effects. This particularly applies to endovascular treatment having been established as standard of care only after RCT data were collected. Further, our real-world dataset was derived from only one registry. Importantly, however, the GSR is one of the most unselective real-world registries^6^ on patients treated with EVT with inclusion criteria limited to age ⩾18 years and the initiation of EVT thus being ideally suited to include the complete spectrum of EVT treated patients. Future studies will show how generalizable our findings are.

In conclusion, we here identified considerable differences between an RCT and a real-world cohort with regards to (i) baseline patient characteristics and procedural metrics, (ii) the relation of these variables to outcome with the most prominent differences seen for age and treatment with thrombolysis, and (iii) overall outcome prediction performance with the real-world cohort-derived machine learning model being most precise. Our findings are important for the interpretation of outcome prediction studies and call for a unified approach of analyses and reporting in future studies.

## Supplemental Material

sj-docx-1-eso-10.1177_23969873221142642 – Supplemental material for RCT versus real-world cohorts: Differences in patient characteristics drive associations with outcome after EVT Click here for additional data file. Supplemental material, sj-docx-1-eso-10.1177_23969873221142642 for RCT versus real-world cohorts: Differences in patient characteristics drive associations with outcome after EVT by Fanny Quandt, Nina Meißner, Teresa A Wölfer, Fabian Flottmann, Milani Deb-Chatterji, Lars Kellert, Jens Fiehler, Mayank Goyal, Jeffrey L Saver, Christian Gerloff, Götz Thomalla and Steffen Tiedt in European Stroke Journal
