# Supplementary material for: RCT versus real-world cohorts: Differences in patient characteristics drive associations with outcome after EVT
Source: Eur Stroke J. 2022 Dec 16;8(1):231–40. doi: 10.1177/23969873221142642 (PMC10069173; doi:10.1177/23969873221142642)
Supplement: sj-docx-1-eso-10.1177_23969873221142642 – Supplemental material for RCT versus real-world cohorts: Differences in patient characteristics drive associations with outcome after EVT [file sj-docx-1-eso-10.1177_23969873221142642.docx]

**SUPPLEMENTAL MATERIAL**

**RCT vs real-world cohorts: differences in patient characteristics drive associations with outcome after endovascular stroke treatment**

Fanny Quandt, MD, Nina Meißner, Teresa Allegra Wölfer, Fabian Flottmann, MD, Milani Deb-Chatterji, MD, Lars Kellert, MD, Jens Fiehler, MD, Mayank Goyal, MD, Jeffrey L Saver, MD, Christian Gerloff, MD, Götz Thomalla, MD, Steffen Tiedt, MD, PhD, on behalf of the GSR investigators and the VISTA-Endovascular Collaborators^†^.

**List of content:**

Supplemental Tables I-IV

Supplemental Figures I-III

**Supplemental Table I: List of GSR investigators**

| **GSR Investigators** | **GSR-ET Center** |
| --- | --- |
| O Nikoubashman, A Reich | Department of Neurology and Department of Neuroradiology, University Hospital RWTH Aachen, Germany |
| J Berrouschot, A Bormann | Department of Neurology and Department of Neuroradiology, Klinikum Altenburger Land, Germany |
| G Bohner, CH Nolte, E Siebert, S Zweynert | Department of Neuroradiology, Institute of Neuroradiology and Department of Neurology, Charite Universitary Medicine Berlin, Germany |
| F Dorn, GC Petzold | Department of Neurology and Department of Neuroradiology, University Hospital Bonn, Germany / German Center for Neurodegenerative Diseases, Bonn, Germany |
| F Keil, W Pfeilschifter | Department of Neurology and Department of Neuroradiology, Institute of Diagnostic and Interventional Neuroradiology, University Hospital Frankfurt, Frankfurt am Main, Germany |
| GF Hamann | Department of Neurology, Bezirkskrankenhaus Günzburg, Germany |
| M Braun | Department of Radiology, Sektion Neuroradiologie Klinik für Diagnostische und Interventionelle Radiologie, Universitätsklinikum Ulm, BKH-Günzburg, Germany |
| A Alegiani, B Eckert, J Röther | Department of Neuroradiology, Institut für Radiologie und Neuroradiologie and Department of Neurology, Neurologische Abteilung, Asklepios Klinik Altona, Hamburg, Germany |
| J Fiehler, C Gerloff, M Schell, G Thomalla | Department of Neurology and Department of Neuroradiology, University Medical Center Hamburg-Eppendorf, Germany |
| C Kraemer | Department of Neurology, Städtisches Klinikum Lüneburg, Germany |
| K Gröschel, T Uphaus | Department of Neurology, University Medical Center of the Johannes Gutenberg University Mainz, Mainz, Germany |
| J Borggrefe, P Schellinger | Universitätsklinik für Neurologie und Radiologie, Mühlenkreiskliniken, Johannes Wesling Klinikum Minden, Universitätsklinikum der Ruhr-Universität Bochum |
| L Kellert, S Tiedt, C Trumm | Department of Neurology, Institute of Neuroradiology, Institute for Stroke and Dementia Researh, University Hospital, LMU Munich, Munich, Germany |
| T Boeckh-Behrens, S Wunderlich | Department of Neuroradiology and Department of Neurology, Clinic and Policlinic for Neurology, Klinikum rechts der Isar, Technical University Munich, Germany |
| KH Henn, A Ludolph | Department of Neurology, Sana Klinikum Offenbach, Germany |
| M Petersen, F Stögbauer | Department of Neurology and Department of Radiology, Klinikum Osnabrück, Germany |
| U Ernemann, S Poli | Department of Neurology With Focus on Neurovascular Diseases, University Hospital Tübingen, Tübingen, Germany |

**Supplemental Table II: List of VISTA-Endovascular Collaborators**

| **VISTA-Endovascular Investigators** | **Affiliation** |
| --- | --- |
| P Khatri (Chair) | Department of Neurology and Rehabilitation Sciences, University of Cincinnati, Cincinnati, OH, USA |
| M Bendszuz | Department of Neuroradiology, Heidelberg University Hospital, Heidelberg, Germany |
| S Bracard | Department of Diagnostic and Interventional Neuroradiology, INSERM U 947, Université de Lorraine and University Hospital of Nancy, France |
| J Broderick | University of Cincinnati Gardner Neuroscience Institute, OH, USA |
| B Campbell | Department of Medicine and Neurology, Melbourne Brain Centre at the Royal Melbourne Hospital, University of Melbourne, Parkville, Australia |
| A Ciccone | Department of Neurology and Stroke Unit, Carlo Poma Hospital, ASST Mantova, Mantua, Italy |
| A Davalos | Department of Neuroscience, Hospital Germans Trias i Pujol, Universitat Autònoma de Barcelona, Barcelona, Spain |
| S Davis | Department of Medicine and Neurology, Melbourne Brain Centre at the Royal Melbourne Hospital, University of Melbourne, Parkville, Australia |
| A Demchuk | Calgary Stroke Program, Departments of Clinical Neurosciences and Radiology, Hotchkiss Brain Institute, Cumming School of Medicine, University of Calgary, Canada |
| HC Diener | Department of Neurology, University Hospital Essen University Duisburg-Essen, Germany |
| D Dippel | Department of Neurology, Erasmus MC University Medical Center, Rotterdam, the Netherlands |
| GA Donnan | The Florey Institute of Neuroscience and Mental Health, University of Melbourne, Parkville, Australia |
| X Ducrocq | Neurology Department, Metz-Thionville Hospital, France |
| J Fiehler | Department of Diagnostic and Interventional Neuroradiology, University Medical Center Hamburg-Eppendorf, Hamburg, Germany |
| D Fiorella | Department of Neurosurgery, Stony Brook University, NY, USA |
| G Ford | Stroke Unit, Oxford University Hospitals and Division of Medical Sciences, Oxford University, United Kingdom |
| M Goyal | Calgary Stroke Program, Departments of Clinical Neurosciences and Radiology, Hotchkiss Brain Institute, Cumming School of Medicine, University of Calgary, Canada |
| W Hacke | Department of Neurology, University of Heidelberg, Heidelberg, Germany |
| M Hill | Calgary Stroke Program, Departments of Clinical Neurosciences, Medicine, Community Health Sciences, and Radiology, Hotchkiss Brain Institute, Cumming School of Medicine, University of Calgary, Canada |
| R Jahan | Division of Interventional Neuroradiology, Department of Radiological Sciences, University of California, Los Angeles (UCLA) |
| E Jauch | Mission Research Institute, Mission Health System, Asheville, NC, USA |
| T Jovin | Department of Neurology, Cooper University Hospital Neurological Institute, Camden, NJ, USA |
| C Kidwell | Department of Radiology, University of Arizona, Tucson, USA |
| KR Lees | Medical School and Institute of Cardiovascular and Medical Sciences, University of Glasgow, Glasgow, UK |
| DS Liebeskind | Department of Neurology, David Geffen School of Medicine at University of California Los Angeles |
| CB Majoie | Department of Radiology, Academic Medical Center Amsterdam, the Netherlands |
| S Martins | Department of Neurology, Federal University of Rio Grande do Sul and Hospital de Clínicas de Porto Alegre, Brazil |
| P Mitchell | Department of Radiology, Royal Melbourne Hospital, University of Melbourne, Parkville, Australia |
| J Mocco | Department of Neurosurgery, Icahn School of Medicine at Mount Sinai, New York City, NY, USA |
| K Muir | Institute of Neuroscience and Psychology, University of Glasgow, Scotland, United Kingdom |
| RG Nogueira | Department of Neurology, Emory University School of Medicine, Marcus Stroke & Neuroscience Center, Grady Memorial Hospital, Atlanta, GA, USA |
| JL Saver | Department of Neurology, David Geffen School of Medicine at the University of California, Los Angeles, Los Angeles, CA, USA |
| WJ Schonewille | Department of Neurology, St. Antonius Hospital, Nieuwegein, the Netherlands |
| AH Siddiqui | Department of Neurosurgery, State University of New York at Buffalo, USA |
| G Thomalla | Department of Neurology, University Medical Center Hamburg-Eppendorf, Hamburg, Germany |
| TA Tomsick | Department of Radiology, University of Cincinnati Academic Health Center, University Hospital, Cincinnati, Ohio, USA |
| AS Turk | Department of Radiology, Medical University of South Carolina, Charleston, USA |
| WH van Zwam | Department of Radiology, Maastricht University Medical Center Maastricht, the Netherlands |
| P White | Institute of Neuroscience, Newcastle University, Newcastle upon Tyne, UK |
| S Yoshimura | Department of Neurosurgery, Hyogo College of Medicine, Nishinomiya, Japan |
| OO Zaidat | Department of Neuroscience, St Vincent Mercy Hospital, Toledo, Ohio, USA |

**Supplemental Table III: List of selection criteria of selected RCTs on endovascular treatment**

| RCT | Selection | Criteria |
| --- | --- | --- |
| MR CLEAN | Inclusion | - **A clinical diagnosis of acute stroke, with a deficit on the NIH stroke scale of 2 points or more.** - CT or MRI scan ruling out intracranial hemorrhage. - **Intracranial arterial occlusion of the distal intracranial carotid artery or middle (M1/M2) or anterior (A1/A2) cerebral artery, demonstrated with CTA, MRA, DSA or transcranial Doppler/duplex (TCD).** - **The possibility to start treatment within 6 hours from onset.** - Informed consent given. - Age 18 or over. |
|  | General exclusion | - Arterial blood pressure > 185/110 mmHg. - Blood glucose < 2.7 or > 22.2 mmol/L. - Intravenous treatment with thrombolytic therapy in a dose exceeding 0.9 mg/kg alteplase or 90 mg. - Intravenous treatment with thrombolytic therapy despite contra‐indications, i.e. major surgery, gastrointestinal bleeding or urinary tract bleeding within the previous 2 weeks, or arterial puncture at a non‐compressible site within the previous 7 days. |
|  | Specific exclusion criteria for intended mechanical thrombectomy | - Laboratory evidence of coagulation abnormalities, i.e. platelet count <40 x 109 /L, APTT>50 sec or INR >3.0 - Cerebral infarction in the distribution of the relevant occluded artery in the previous 6 weeks. - History of intracerebral hemorrhage. - Severe head injury (contusion) in the previous 4 weeks. - Clinical or laboratory evidence of coagulation abnormalities, i.e. platelet count <90 x 109 /L, APTT>50 sec or INR >1.7 |
| ESCAPE | Inclusion | - Acute ischemic stroke - Age 18 or greater - **Onset (last‐seen‐well) time to randomization time < 12 hours.** - **Disabling stroke defined as a baseline NIHSS > 5 at the time of randomization.** - **Pre‐stroke (24 hours prior to stroke onset) independent functional status in activities of daily living with modified Barthel Index > 90. Patient must be living in their own home, apartment or seniors lodge where no nursing care is required.** - **Confirmed symptomatic intracranial occlusion, based on single phase, multiphase or dynamic CTA, at one or more of the following locations: Carotid T/L, M1 MCA, or M1‐MCA equivalent (2 or more M2‐MCAs). Anterior temporal artery is not considered an M2.** - Non‐contrast CT and CTA for trial eligibility performed or repeated at ESCAPE stroke center with endovascular suite on‐site. - Endovascular treatment intended to be initiated (groin puncture) within 60 minutes of baseline non‐contrast CT with target baseline non‐contrast CT to first recanalization of 90 minutes. - Signed informed consent or appropriate signed deferral of consent where approved. |
|  | Exclusion | - **Baseline non‐contrast CT reveals a moderate/large core defined as extensive early ischemic changes of ASPECTS 0‐5 in the territory of symptomatic intracranial occlusion.** - Other confirmation of a moderate to large core defined one of three ways:   - On a single phase, multiphase or dynamic CTA: no or minimal collaterals in a region greater than 50% of the MCA territory when compared to pial filling on the contralateral side (multiphase/dynamic CTA preferred)   - On CT perfusion (>8 cm coverage): a low CBV and very low CBF ASPECTS <6 AND in the symptomatic MCA territory   - On CT perfusion(<8 cm coverage): a region of low CBV and very low CBF >1/3 of the CTP imaged symptomatic MCA territory. - Groin puncture is not possible within 60 minutes of the first slice of non‐contrast CT acquisition (please note that if CTP is performed it should be done after CTA). - No femoral pulses or very difficult endovascular access that will result in a non‐contrast CT‐to‐recanalization time that is longer than 90 minutes, or will result in an inability to deliver endovascular therapy. - Pregnancy; if a woman is of child‐bearing potential a urine or serum beta HCG test is positive. - Severe contrast allergy or absolute contraindication to iodinated contrast. - Suspected intracranial dissection as a cause of stroke. - Clinical history, past imaging or clinical judgment suggests that the intracranial occlusion is chronic. - Patient has a severe or fatal comorbid illness that will prevent improvement or follow‐up or that will render the procedure unlikely to benefit the patient. |
| SWIFT-PRIME | Inclusion | - **Age 18 – 85** - Clinical signs consistent with acute ischemic stroke - **No prestroke functional dependence (prestroke Modified Rankin Score ≤ 1)** - **NIHSS ≥ 8 and < 30 at the time of randomization** - **Initiation of IV t-PA within 4.5 hours of onset of stroke symptoms (onset time is defined as the last time when the patient was witnessed to be at baseline), with investigator verification that the subject has received / is receiving the correct IV t-PA dose for the estimated weight prior to randomization.** - **Thrombolysis in Cerebral Infarction (TICI) 0-1 flow in the intracranial internal carotid artery, M1 segment of the MCA, or carotid terminus confirmed by CT or MR angiography that is accessible to the Solitaire™ FR Device.** - **Subject is able to be treated (with minimum 1 deployment of Solitaire™ FR Device) within 1.5 hours of CTA/PCT or PWI/MRA MRI.** - Subject is willing to conduct protocol-required follow-up visits. - Subject or subject’s legally authorized representative has signed and dated an Informed Consent Form according to country regulations, ethics committee, and/or IRB requirements. |
|  | Exclusion | - History of stroke in the past 3 months. - Female who is pregnant or lactating or has a positive pregnancy test at time of admission. - Rapid neurological improvement prior to study randomization suggesting resolution of signs/symptoms of stroke. - Known serious sensitivity to radiographic contrast agents. - Current participation in another investigational drug or device treatment study. - Uncontrolled hypertension defined as systolic blood pressure > 185 or diastolic blood pressure > 110 that cannot be controlled except with continuous parenteral antihypertensive medication. - Known hereditary or acquired hemorrhagic diathesis, coagulation factor deficiency. (Patients without history or suspicion of coagulopathy do not require INR or prothrombin time lab results to be available prior to enrollment.) - Warfarin therapy with INR greater than 1.7. - Low molecular Weight Heparins (such as Dalteparin, Enoxaparin, Tinzaparin, Fondaparinux) as DVT prophylaxis or in full dose within the last 24 hours from screening. - Subject who has received heparin or a direct thrombin inhibitor (e.g. rivaroxaban, Angiomax™, argatroban, Refludan™) within the last 48 hours must have a normal partial thromboplastin time (PTT) to be eligible. - Subject who has received factor Xa inhibitor therapy (e.g. dabigatran) within the past 24 hours must have a normal ecarin clotting time to be eligible. Subject who has received factor Xa inhibitor therapy more than 24 hours ago but less than 48 hours ago must have a normal partial thromboplastin time (PTT) to be eligible. - Baseline lab values: glucose < 50 mg/dl or > 400 mg/dl, platelets < 100,000, or Hct < 25 - Renal Failure as defined by a serum creatinine > 2.0 or Glomerular Filtration Rate [GFR] < 30. - Subject who requires hemodialysis or peritoneal dialysis, or who have a contraindication to an angiogram for whatever reason. - Life expectancy of less than 90 days - Previous intra-cranial hemorrhage, neoplasm, subarachnoid hemorrhage, cerebral aneurysm, or arteriovenous malformation - Clinical presentation suggests a subarachnoid hemorrhage, even if initial CT or MRI scan is normal. - Presumed septic embolus, or suspicion of bacterial endocarditis. - Presumed pericarditis including pericarditis after acute myocardial infarction. - Suspicion of aortic dissection - Surgery or biopsy of parenchymal organ within 30 days. - Trauma with internal injuries or ulcerative wounds within 30 days. - Severe head trauma or head trauma with loss of consciousness within 90 days. - Any active or recent hemorrhage within 30 days. - Cerebral vasculitis - Subject with a pre-existing neurological or psychiatric disease that would confound the neurological and functional evaluations. |
|  | Imaging Exclusion Criteria | - Computed tomography (CT) or Magnetic Resonance Imaging (MRI) evidence of hemorrhage on presentation - CT showing hypodensity or MRI showing hyperintensity involving greater than 1/3 of the middle cerebral artery (MCA) territory (or in other territories, >100 cc of tissue) on presentation - CT or MRI evidence of mass effect or intra-cranial tumor (except small meningioma) - Core Infarct and hypoperfusion:   - MRI-assessed core infarct lesion greater than:     - 50 cc for subjects age 18-79 years;     - 20 cc for subjects age 80-85 years;   - CT-assessed core infarct lesion greater than:     - 40 cc for subjects age 18-79 years;     - 15 cc for subjects age 80-85 years;   - All subjects, severe hypoperfusion lesion (10 sec or more Tmax lesion larger than 100 cc);   - All subjects, ischemic penumbra ≥ 15 cc and mismatch ratio >1.8. - Angiographic evidence of carotid dissection or complete cervical carotid occlusion - Arterial tortuosity, calcification, pre-existing stent, and/or stenosis which would prevent the device from reaching the target vessel and/or preclude safe recovery of the device |
| EXTEND-IA | Inclusion | - Patients presenting with anterior circulation acute ischemic stroke eligible using standard criteria to receive IV tPA within 4.5 hours of stroke onset - Patient, family member or legally responsible person depending on local ethics requirements has given informed consent - Patient’s age is ≥18 years - **Intra-arterial clot retrieval treatment can commence (groin puncture) within 6 hours of stroke onset.**   Imaging inclusion criteria: Dual target:   - Arterial occlusion on CTA or MRA of the ICA, M1 or M2 - Mismatch - Using CT or MRI with a Tmax >6 second delay perfusion volume and either CT-rCBF or DWI infarct core volume.   - Mismatch ratio of greater than 1.2, and   - Absolute mismatch volume of greater than 10 ml, and   - Infarct core lesion volume of less than 70mL |
|  | Exclusion | - Intracranial hemorrhage (ICH) identified by CT or MRI - Rapidly improving symptoms at the discretion of the investigator - **Pre-stroke mRS score of ≥ 2 (indicating previous disability)** - Hypodensity in >1/3 MCA territory on non-contrast CT - Inability to access the cerebral vasculature in the opinion of the neurointervention team - Stenosis proximal to the thrombus site that may preclude safe recovery of the Solitaire™ FR stent retriever - Angiographic evidence of carotid dissection - Contra indication to imaging with contrast agents - Known hypersensitivity to nickel-titanium - Participation in any investigational study in the previous 30 days - Any terminal illness such that the patient would not be expected to survive more than 1 year - Any condition that, in the judgment of the investigator could impose hazards to the patient if study therapy is initiated or affect the participation of the patient in the study - Pregnant women - Previous stroke within last three months - Recent past history or clinical presentation of ICH, subarachnoid hemorrhage (SAH), arterio-venous (AV) malformation, aneurysm, or cerebral neoplasm (at the discretion of each Investigator) - Current use of oral anticoagulants and a prolonged prothrombin time (INR > 1.7) - Use of heparin, except for low dose subcutaneous heparin, in the previous 48 hours and a prolonged activated partial thromboplastin time exceeding the upper limit of the local laboratory normal range. - Use of glycoprotein IIb-IIIa inhibitors within the past 72 hours. Prior use of single or dual agent oral platelet inhibitors (clopidogrel and/or low-dose aspirin) permitted - Clinically significant hypoglycaemia - Uncontrolled hypertension defined by a blood pressure > 185 mmHg systolic or >110 mmHg diastolic on at least 2 separate occasions at least 10 minutes apart, or requiring aggressive treatment to reduce the blood pressure to within these limits (the definition of “aggressive treatment” is left to the discretion of the responsible Investigator) - Hereditary or acquired hemorrhagic diathesis - Gastrointestinal or urinary bleeding within the preceding 21 days - Major surgery within the preceding 14 days which posed risk in the opinion of the Investigator |
| REVASCAT | Inclusion | - Acute ischemic stroke where patient is ineligible for IV thrombolytic treatment or the treatment is contraindicated (e.g., subject presents beyond recommended time from symptom onset), or where patient has received IV thrombolytic therapy without recanalization after a minimum of 30 min from start of iv tPA infusion - **No significant pre-stroke functional disability (mRS ≤ 1)** - **Baseline NIHSS score obtained prior to randomization must be equal or higher than 6 points** - **Age ≥18 and ≤ 80** - Occlusion (TICI 0-1) of the intracranial ICA (distal **ICA or T occlusions), MCA-M1 segment or tandem proximal ICA/MCA-M1 suitable for endovascular treatment, as evidenced by CTA, MRA or angiogram, with or without concomitant cervical carotid occlusion or stenosis** - **Patient treatable within eight hours of symptom onset. Symptoms onset is defined as point in time the patient was last seen well (at baseline). Treatment start is defined as groin puncture.** - Informed consent obtained from patient or acceptable patient surrogate |
|  | Exclusion | Clinical criteria   - Known hemorrhagic diathesis, coagulation factor deficiency, or oral anticoagulant therapy with INR > 3.0 - Baseline platelet count < 30.000/μL - Baseline blood glucose of < 50mg/dL or >400mg/dl - Severe, sustained hypertension (SBP > 185 mm Hg or DBP > 110 mm Hg) NOTE: If the blood pressure can be successfully reduced and maintained at the acceptable level using ESO guidelines recommended medication (including iv antihypertensive drips), the patient can be enrolled. - Patients in coma (NIHSS item of consciousness >1) (Intubated patients for transfer could be randomized only in case an NIHSS is obtained by a neurologist prior transportation). - Seizures at stroke onset which would preclude obtaining a baseline NIHSS - Serious, advanced, or terminal illness with anticipated life expectancy of less than one year. - History of life threatening allergy (more than rash) to contrast medium - Subjects who has received iv t-PA treatment beyond 4,5 hours from the beginning of the symptoms - Renal insufficiency with creatinine ≥ 3 mg/dl - Woman of childbearing potential who is known to be pregnant or lactating or who has a positive pregnancy test on admission. - Subject participating in a study involving an investigational drug or device that would impact this study. - Cerebral vasculitis - Patients with a pre-existing neurological or psychiatric disease that would confound the neurological or functional evaluations, mRS score at baseline must be ≤ 1. This excludes patients who are severely demented, require constant assistance in a nursing home type setting or who live at home but are not fully independent in activities of daily living (toileting, dressing, eating, cooking and preparing meals, etc.) - Unlikely to be available for 90-day follow-up (e.g. no fixed home address, visitor from overseas).   Neuroimaging criteria   - **Hypodensity on CT or restricted diffusion amounting to an ASPECTS score of <7 on CT, CTP-CBV, CTA-SI or <6 on DWI MRI. ASPECTS must be evaluated by CBV maps of CT Perfusion, CTA source imaging (CTA-SI) or DWI-MR in patients whose vascular occlusion study (CTA/MR) confirming qualifying occlusion, is performed beyond 4.5 hours of last seen well**. - CT or MR evidence of hemorrhage (the presence of microbleeds is allowed). - Significant mass effect with midline shift. - Evidence of ipsilateral carotid occlusion, high grade stenosis or arterial dissection in the extracranial or petrous segment of the internal carotid artery that cannot be treated or will prevent access to the intracranial clot or excessive tortuosity of cervical vessels precluding device delivery/deployment - Subjects with occlusions in multiple vascular territories (e.g., bilateral anterior circulation, or anterior/posterior circulation) - Evidence of intracranial tumor (except small meningioma). |

Bolded selection criteria indicate criteria that (i) are based on information available in the GSR and (ii) differ from selection criteria of the GSR.

**Supplemental Table IV: Characteristics of patients with anterior circulation LVO stroke that underwent EVT from the German Stroke Registry and from VISTA-Endovascular within 6 hours from symptom onset**

| **Characteristics** | **GSR**  N=2,772 | **VISTA**  N=446 | *P* value |
| --- | --- | --- | --- |
| Age, median (IQR) [years] | 75 (64-81) | 68 (57-77) | <0.0001 |
| Female, N (%) | 1,337 (48.2) | 202 (45.3) | 0.261 |
| Medical history, N (%) |  |  |  |
| Hypertension | 2,104 (75.9) | 244 (54.7) | <0.0001 |
| Diabetes mellitus | 566 (20.4) | 71 (15.9) | 0.029 |
| Dyslipidemia | 1,099 (39.6) | 127 (28.5) | <0.0001 |
| Current smoking | 418 (15.1) | 127 (28.5) | <0.0001 |
| Pre-stroke mRS score, median (IQR) | 0 (0-0) | 0 (0-0) | 0.030 |
| Baseline NIHSS score, median (IQR) | 15 (11-19) | 17 (14-20) | <0.0001 |
| ASPECTS, N (%) |  |  | 0.073 |
| High (9-10) | 1,343 (48.4) | 242 (54.3) |  |
| Middle (6-8) | 920 (33.2) | 177 (39.7) |  |
| Low (0-5) | 229 (8.3) | 27 (6.1) |  |
| Intravenous thrombolysis, N (%) | 2,187 (59.6) | 383 (85.9) | <0.0001 |
| Symptom onset to arterial puncture, median (IQR) [min] | 214 (150-310) | 229 (169-285) | 0.078 |
| mRS score 0-2 at 90 days after stroke, N (%) | 1,219 (44.0) | 208 (46.6) | 0.304 |

IQR, interquartile range.

**Supplementary Figure 1**


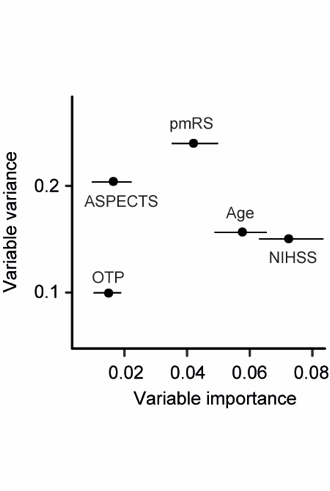


**Variable importance for outcome prediction is not related to variable variance.** Importance values of continuous variables for predicting functional outcome at 90 days of patients with anterior LVO stroke (GSR dataset) plotted against the variance of these variables. Variances were determined by calculating the standard deviation of variables after rescaling them from 0 to 1. LVO, large vessel occlusion; GSR, German Stroke Registry; pmRS, premorbid modified Rankin Scale; ASPECTS, Alberta Stroke Program Early CT Score; OTP, onset-to-puncture time; NIHSS, National Institutes of Health Stroke Scale.

**Supplementary Figure 2**


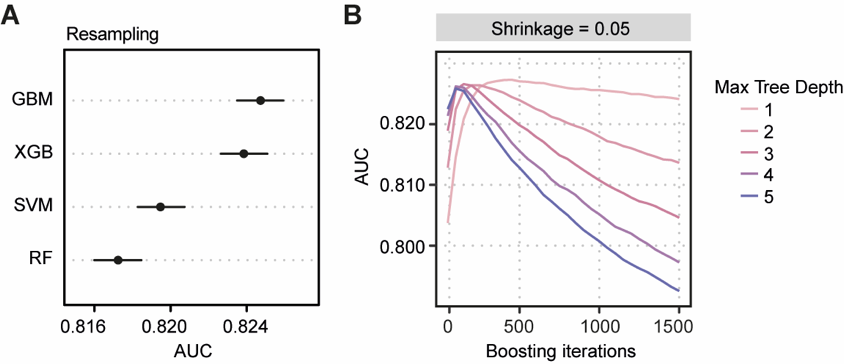


**Identification and optimization of the machine learning algorithm.** A: Gradient boosting machines performed slightly better than other machine learning classifiers in predicting functional independence at 90 days in patients with LVO stroke in the anterior circulation that underwent EVT. B: Examplary illustration of GBM hyperparameter optimization (Number of trees = 350, depth of interaction = 1). A and B: same dataset from the German Stroke Registry. GBM, gradient boosting machine; XGB, extreme gradient boosting; SVM, support vector machine; RF, random forest.

**Supplementary Figure 3**


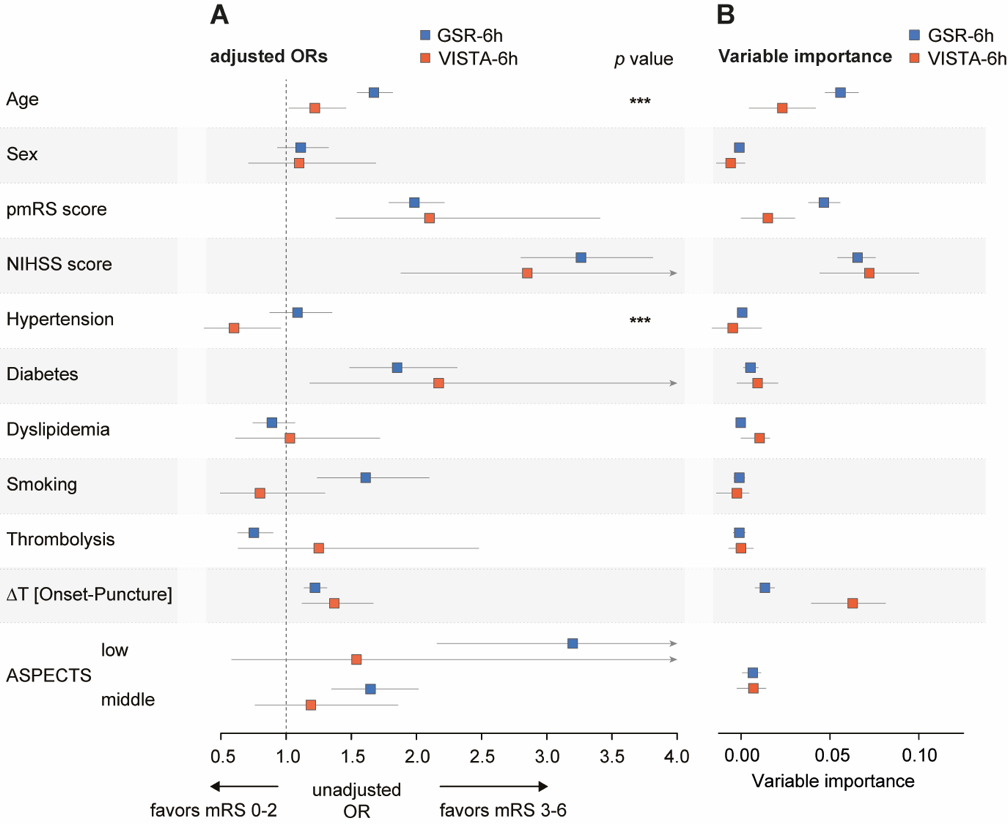


**Association with and variable importance for the prediction of outcome in patients treated within 6 hours from symptom onset.** A: Age and hypertension significantly differed in their relation to functional outcome between both cohorts in adjusted analyses. B: Age, the pmRS score, and onset-to-puncture time showed different variable importance for predicting outcome between both cohorts. A: Multivariable logistic regression analyses adjusted for all other variables. Odds ratios were calculated for age per 10-year increments, for female vs male sex, for the pmRS score in one-point increments, for the NIHSS score in 10-point increments, for hypertension, diabetes, and dyslipidemia with yes vs no, for smoking with any history of smoking vs no history, for thrombolysis with treatment vs no treatment, for the time from onset to puncture per 1-hour delay, and for ASPECTS with low (0-5) and middle (6-8) vs high. P values indicate the levels of significance from the interaction term for investigating whether cohort assignment (RCT vs RWD) introduces heterogeneity and are indicated as follows: * for p < 0.05, ** for p < 0.01, *** for p < 0.001, **** for p < 0.0001. B: Variable importance analyses to calculate the contribution of each parameter to outcome prediction using a machine learning algorithm (gradient boosting machines). Boxes indicate the median and whiskers the 5 % and 95 % quantiles. A & B: Data from the GSR cohort (N=4,077), the VISTA cohort (N=479). GSR, German Stroke Registry; OR, odds ratio; pmRS, premorbid modified Rankin Scale; ASPECTS, Alberta Stroke Program Early CT Score; ∆T, time difference; NIHSS, National Institutes of Health Stroke Scale.
